# Editorial: Molecular drivers of prostate cancer pathogenesis and therapy resistance

**DOI:** 10.3389/fcell.2023.1239478

**Published:** 2023-06-23

**Authors:** Girijesh Kumar Patel, Santosh Kumar Verma, Shagun Misra, Gyan Chand, Ram Nawal Rao

**Affiliations:** ^1^ Department of Gastroenterology, Sanjay Gandhi Postgraduate Institute of Medical Sciences, Lucknow, India; ^2^ Department of Molecular Medicine and Biotechnology, Sanjay Gandhi Postgraduate Institute of Medical Sciences, Lucknow, India; ^3^ Department of Radiotherapy, Sanjay Gandhi Postgraduate Institute of Medical Sciences, Lucknow, India; ^4^ Department of Endocrine Surgery, Sanjay Gandhi Postgraduate Institute of Medical Sciences, Lucknow, India; ^5^ Department of Pathology, Sanjay Gandhi Postgraduate Institute of Medical Sciences, Lucknow, India

**Keywords:** castrate-resistant prostate cancer, metastasis, neuroendocrine differentiation, cancer stem cell, therapy resistance, recurrence

Prostate cancer (PCa) is the most diagnosed malignancies in the men worldwide (Sung et al., 2021). Blood PSA level (>2.5–4 ng/mL) is primarily used to screen PCa in men with or without symptoms (David and Leslie, 2023). However, elevated PSA level does not confirm the incidence of PCa as other conditions such as prostatitis or benign prostatic hyperplasia (BPH) also show elevated level of PSA. Therefore, additional tests such as digital rectal exam, multiparametric MRI/transrectal ultrasound, biopsy-based cytopathology/histopathology/Immunohistochemical (IHC) analysis are commonly used to confirm the incidence of PCa (David and Leslie, 2023). Androgen deprivation therapy (ADT) remains the standard of care for PCa patients. Notwithstanding an initial favorable response, the majority of the PCa patients invariably progress to castrate-resistant prostate cancer (CRPC). Currently, several hormonal and non-hormonal therapeutic agents including enzalutamide, abiraterone acetate, cabazitaxel, darolutamide, apalutamide, sipuleucel-T, Olaparib, radium-223 are being used to treat PCa (Patel et al., 2019; Verma et al., 2023). These agents showed significant survival benefits in patients with metastatic or non-metastatic CRPC while other promising agents are under clinical trial (Verma et al., 2023). Despite these advancements, PCa remains 2nd most common cause of cancer-related death in men (Sung et al., 2021). Therefore, understanding the underlying molecular mechanisms and identification of effective therapeutic targets are very crucial to manage the PCa in the clinics.

Androgen receptor (AR) signaling regulates several vital pathways. However, aberrant AR signaling leads to multiple oncogenic events including cellular proliferation, migration, invasion, differentiation and cell survival (Kim et al., 2022; Srivastava et al., 2022; Dutta et al., 2023). In recent years, several molecular drivers of PCa pathogenesis and therapy resistance are identified (Testa et al., 2019; Verma et al., 2023). Variants of AR (AR-Vs) (Antonarakis et al., 2014), AR alternate signaling pathways (glucocorticoid receptor signaling) (Puhr et al., 2018), TMPRSS2-ERG fusion (Demichelis et al., 2007), loss of PTEN (Whang et al., 1998), RB1 and P53 (Mu et al., 2017) are well established molecules which play critical role in PCa pathogenesis and therapy resistance. It is demonstrated that MYCN and AURKA cooperate to induce NEPC (Beltran et al., 2011). MYCN also shown to induce EZH2-mediated transcriptional programming to drive NEPC (Dardenne et al., 2016). Dual loss of *TP53* and *RB1* are shown to promote SOX2-mediated lineage plasticity and ADT resistance (Mu et al., 2017). Using genetically engineered mouse models and correlative human studies, NSD2 was identified as a cell-intrinsic drivers of metastatic PCa (Aytes et al., 2018). It is reported that Wnt-signaling regulates prostatic growth and FOXA2 expression during NEPC development and the loss of FOXA2 is compensated by upregulation of pro-neural gene (*Mash1*) in TRAMP mouse model (Gupta et al., 2013)*.* Furthermore, NEPC-secreted neuropeptides (gastrin releasing peptide (GRP) and bombesin) regulate NF-кB mediated regulation of AR-V7 and promote CRPC (Jin et al., 2008). Our group showed the role of MYB-AR crosstalk in stemness and ADT resistance (Srivastava et al., 2022). In the absence of androgen, MYB overexpressing PCa cells sustained AR transcriptional activity and retained AR in the nucleus leading to CRPC (Srivastava et al., 2022). Nandana et al. (2017) established the role of TBX2 in bone metastasis through WNT signaling in PCa. In addition, they further demonstrated that TBX2 by suppressing miR-200c-3p via cell-intrinsic and exosome-mediated paracrine manners promotes SOX2 and N-MYC mediated neuroendocrine differentiation (Patel et al., 2021). Recently, they further identified TBX2 suppression of AR results in GR overexpression and enzalutamide resistance (Dutta et al., 2023). Similarly, Zhang et al. (2020) identified that loss of CHD1 leads to overexpression of oncogenic transcription factors (NR3C1, POU3F2, NR2F1, and TBX2) in which TBX2 was shown to involve in antiandrogen resistance. Altogether, these molecules are shown to drive PCa pathogenesis, metastasis, stemness, NEPC, therapy resistance and cancer relapse. Notwithstanding all these identified molecular drivers and current therapeutic options, many questions remain unanswered regarding the PCa progression and resistance. Therefore, further research is required to identify effective molecular targets for the clinical management of the disease.

Metabolic adaptation of cancer cells dormancy is known to play an important role in therapy resistance. A study by Bort et al. showed metabolic fingerprinting of chemotherapy-resistant PCa stem cells using LC-MS and other approaches, and identified repressed fatty acid oxidation, methionine metabolism and ADP-ribosylation pathways promoting the entry of PCa cells into dormancy (Bort et al.). Dormant cancer cells remain occult, asymptomatic, and resistant to therapy and thus thought to be a major cause of cancer relapse.

Identification of novel molecules for early detection of PCa is highly desirable. Vahabzadeh et al. constructed a lncRNA-miRNA-mRNA network and analyzed to develop potential predictive biomarkers. They identified differential expression four lncRNAs (NEAT1, MALAT1, PCAT19, and CASC2), five miRNAs and 15 common target genes. Among them, oncogenic ALB, APOE, F2, and FAP were significantly upregulated and tumor suppressor such as BDNF, MET, PLG, MMP1, ITGA6, ITGA5, FGF18, CD44, CXCL12, IL10, and ITGB3, were significantly downregulated in PCa patients compared to the healthy control. The interactions between lncRNAs, miRNAs, and mRNAs could be utilized to develop novel biomarkers for assessing treatment response in PCa patients.

Identification of different genetic mutations (Such as DPYD, BRCA1, and BRCA2/HER2) are utilized for the prediction of drug response. Zhao et al. represented a case report of a 65 year old PCa patients which was treated with multiple treatment regimens. Patient’s response was significant when treated with cisplatin+paclitaxelfollowed by Nilaparib combined with endocrine therapy. After 9 months of Nilaparib maintenance therapy, the disease further progressed. Therefore further treated with docetaxel+cisplatin regimen showed poor response and disease progressed. Genetic testing identified TP53, BRCA1, and BRCA2 gene mutations underscoring the role of genetic mutations in therapy response.

Zhu et al. used the publicly available database and identified differentially expressed genes in PCa. Among them, MYLK, MYL9, MYH11, CALD1, ACTA2, SPP1, and CNN1 are identified as hub genes which were associated with proliferation, invasion, and migration of PCa cells and promoting tumor neovascularization which may serve as prognostic markers and therapeutic targets for PCa patients.

Together, the evidence summarized in this topical Research Topic highlights how these articles provide new insights about several molecules and their interactions that play important role in mediating PCa progression, metastasis, and therapy resistance. These studies further suggest that metabolic adaptation, lncRNA-miRNA-mRNA network analysis, identification of hub genes associated with PCa occurrence, and genetic testing may be helpful to decision making to treat PCa. Such studies may apply to achieve early diagnosis, understanding the metastatic progression and designing effective treatment strategies for better clinical outcomes.
